# Exploring Antimicrobial Compounds from Agri-Food Wastes for Sustainable Applications

**DOI:** 10.3390/ijms252313171

**Published:** 2024-12-07

**Authors:** Mattia Di Maro, Luca Gargiulo, Giovanna Gomez d’Ayala, Donatella Duraccio

**Affiliations:** 1 Institute of Sciences and Technologies for Sustainable Energy and Mobility (STEM), National Research Council, Strada delle Cacce 73, 10135 Torino, Italy; mattia.dimaro@stems.cnr.it (M.D.M.); donatella.duraccio@stems.cnr.it (D.D.); 2 Institute of Polymers, Composites and Biomaterials (IPCB), National Research Council, Via Campi Flegrei, 34, 80078 Pozzuoli, Italy; luca.gargiulo@ipcb.cnr.it

**Keywords:** extraction, agri-food wastes, sustainability, phenolic compounds, essential oils, antimicrobial properties

## Abstract

Transforming agri-food wastes into valuable products is crucial due to their significant environmental impact, when discarded, including energy consumption, water use, and carbon emissions. This review aims to explore the current research on the recovery of bioactive molecules with antimicrobial properties from agri-food waste and by-products, and discusses future opportunities for promoting a circular economy in its production and processing. Mainly, antibacterial molecules extracted from agri-food wastes are phenolic compounds, essential oils, and saponins. Their extraction and antimicrobial activity against a wide spectrum of bacteria is analyzed in depth. Also, their possible mechanisms of activity are described and classified based on their effect on bacteria, such as the (i) alteration of the cell membrane, (ii) inhibition of energy metabolism and DNA synthesis, and iii) disruption of quorum sensing and biofilm formation. These bioactive molecules have a wide range of possible applications ranging from cosmetics to food packaging. However, despite their potential, the amount of wastes transformed into valuable compounds is very low, due to the high costs relating to their extraction, technical challenges in managing supply chain complexity, limited infrastructure, policy and regulatory barriers, and public perception. For these reasons, further research is needed to develop cost-effective, scalable technologies for biomass valorization.

## 1. Introduction

Global society is making significant efforts to adopt a comprehensive approach to shift from a linear to a circular economy, implementing various initiatives to reduce the vast amounts of waste generated each year. The continuous growth of world population and the consequent increase in food production lead to the generation of large volumes of waste throughout the whole supply chain, from cultivation and harvest to distribution and consumption. Several agro-industrial processes generate substantial by-products or residues, significantly impacting the environment, production costs, and human health and well-being. Agri-food waste encompasses all agricultural and food products that enter the production cycle but fail to reach consumers’ tables. This includes food lost or discarded at any stage of the supply chain, as well as food purchased for home or catering use that remains uneaten. It is estimated that approximately one-third to half of all food produced globally ends up being wasted [1]. Hundreds of millions of tons of fruit and vegetable waste are discarded worldwide every year, resulting in the loss of valuable reusable resources and contributing heavily to environmental pollution [2,3].

Agricultural production accounts for more than two-thirds of global food waste [4]. Typically, this waste is dealt with through methods like landfilling, incineration, and composting [5]. In Europe, food waste comprises over 50% of municipal solid waste [6,7]. This pervasive waste highlights the urgent need for interventions to reduce and upcycle wastes across the entire agri-food supply chain. The global market potential for upcycling food waste is valued at approximately USD 700 billion annually [8].

The fate of wasted food is still predominantly represented by landfill. Unfortunately, the share of food recovered, recycled, and reused in other forms is still very small. The large amount of organic waste that ends up in landfills also contributes to the production of greenhouse gases, especially methane, further aggravating the problem of climate change. Less is recovered for compost or animal feed. Agri-food companies increasingly aim to increase their capacity to respond to a demand for environmental sustainability and resilience at the same time, and they need to innovate business models thanks also to the role and the impetus that comes from start-ups focusing on sustainability issues. Thanks to start-ups, the agri-food chain can find new resources to transform difficulties and critical situations into opportunities for sustainable development. The organic fraction collection and processing chain, both at the level of municipal waste and industrial waste, is flexible as it can be treated both at the industrial and local community level; generates environmental benefits, i.e., reduction in waste to landfill, biogas (renewable energy), compost (can be used as an alternative fertilizer to industrial products and better tolerated by the environment); and contributes to combating climate change (both in terms of reducing climate-related emissions and by producing organic substances, which can be used to restore degraded or desertified land). Moreover, this approach is economically sustainable: waste management can be implemented at company installations directly on-site, generating electricity and reusable raw materials in the same farm or at short distance away and managed locally. Furthermore, collection chains for end-product use are highly suitable for local management by reducing pollutant emissions from supply chains.

**Figure 1:**
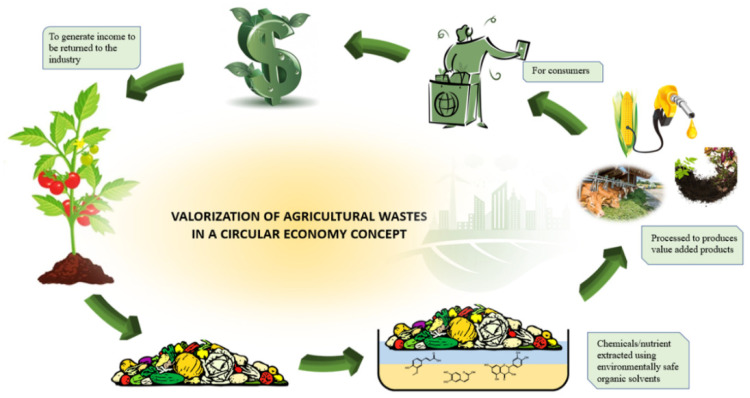
The application green principles to valorize agricultural waste within a circular economy framework. Reproduced from [11].

Agricultural and food wastes represent lost resources, as they contain an abundance of natural polymers such as polysaccharides, proteins, and lignin [9]. Moreover, in biorefineries, they are considered as optimal feedstocks due to their economic advantages, such as low transportation and storage costs, constant year-round availability, and ease of processing [10]. Transforming these wastes into valuable products is crucial due to their significant environmental impact, including energy consumption, water use, and carbon emissions, when discarded. The ultimate aim is to ensure that all by-products from agri-food production and processing are converted into useful resources, aligning with the principles of upcycling and a circular bioeconomy (Figure 1) [11]. This review explores the current research on the sources of food and agricultural waste and by-products, outlines recent progress in waste reduction and value-added utilization, and discusses future opportunities for promoting a circular economy in agri-food production and processing, with special emphasis on the recovery of bioactive molecules with antimicrobial properties. In the following paragraphs, a general classification of the recoverable components from agri-food wastes will be reported. Then, the attention will be focused on bioactive compounds with antimicrobial activity, on the different extraction methods and on the main results on antimicrobial metabolites and their applications. A paragraph describing the possible mechanisms of antimicrobial will also be included. Finally, an overview of future perspective for the valorization of the molecules extracted from agri-food waste will be provided.

## 2. Classification and Properties of Recoverable Components from Agri-Food Wastes 

Agri-food wastes represent a valuable source of numerous recoverable components that can be classified into several categories based on their structural properties, functionalities and potential applications, i.e., biomaterials (biopolymers) and bioactive compounds. 

Among renewable biopolymers, there are natural polysaccharides (e.g., cellulose, pectin, starch and β-glucans), which represent a massive part of the waste biomasses. They can be recovered and utilized as sustainable alternative to traditional petroleum-based plastics across various applications. 

Each class of biopolymers exhibits peculiar physicochemical properties, making agri-food waste a versatile resource for sustainable applications. Their renewable nature, along with features like biocompatibility and degradability, helps reduce overall waste and fosters a circular economy approach.

Razzaq et al. extracted mixtures based on β-glucans and proteins from barley grains in mild and high alkaline conditions, through a very sustainable and cost-effective method. The mixture was used to prepare films through the solvent casting method, with both composition and physico-chemical properties strictly dependent on extraction conditions. The outstanding mechanical properties, inherent antibacterial activity against both Gram+ and Gram− bacteria, along with the promising anti-inflammatory properties, make them highly suitable as bioactive wound dressings for biomedical applications [12,13]. Furthermore, among polysaccharides, pectins represent some of the main components of the primary cell walls of intracellular surfaces of apples, oranges, citrus fruits. They can be extracted from waste biomasses by using mild organic solvents, and due to their biodegradability and biocompatibility, along with their gelling capability, can be employed as a polymer matrix for different applications. Quilez-Molina et al. developed starch composites containing pectin-based extracts from orange peel with excellent mechanical properties and moisture resistance [14]. 

Proteins exhibit structural properties that enhance their functionalities in various industries. Their diverse amino acid profiles and robust structural characteristics contribute to improved stability, emulsification, and foaming capabilities, making them valuable in food, pharmaceutical, and biotechnological applications [15,16].

Potato proteins (i.e., patatin, protease inhibitors, and different high-molecular-weight proteins) can be sourced from potato starch production, peels, and damaged potatoes. Rich in hydrophobic amino acids with aromatic side chains, these proteins can be employed as food additives and in bioplastic production. Films obtained from potato protein flours, through thermoforming or compression molding, exhibit good mechanical properties, while cast films demonstrate significant barrier properties, showcasing their potential in the packaging industry [15].

Agricultural residues are also a source of large quantities of lignocellulose that can be recovered through different methodologies. Lignocellulose, embedded in the plant cell matrix, forms a composite of cellulosic tissues primarily composed of polysaccharides (cellulose and hemicellulose) and lignin, which has inherent antimicrobial activity against various microorganisms. It also includes minor components like pectin, proteins, salts, and minerals, interconnected by hydrogen and covalent bonds, creating a highly recalcitrant structure [17]. The primary application of this component is the reinforcement of polymers to create new, high-performance composites for various purposes [18]. Zannini et al. exploited the lignocellulose fraction extracted from citrus pomace, along with pectin, to develop biocomposites to be used as biodegradable mulch systems for protecting crops [19]. Goswami et al. extracted cellulose nanocrystals from sugarcane bagasse fibers and mixed them with alginate to develop composite beads for the removal of chromium ions from wastewater. 

Bioactive compounds from agri-food wastes include a wide range of valuable secondary metabolites with promising functional properties, such as antioxidants, antimicrobials, and anti-inflammatory activities. This class encompasses phenolic compounds such as flavonoids, tannins, stilbenes, saponins, and essential oils (EOs). Among them, phenolic compounds represent the main class of antibacterial molecules extracted from agri-food wastes and possess multiple healthy properties, including antioxidant, anti-inflammatory, and hypolipidemic activities [20]. They consist of simple phenols, benzoic and cinnamic acid, coumarins, tannins, lignins, lignans, and flavonoids, and are well-known phytochemicals found in all plants.

EOs consist of lipophilic and highly volatile organic compounds with a molecular mass generally below 300 Da. They can be extracted from various parts of plants (i.e., flowers, leaves, stems, roots, bark, and fruits) through mechanical or physical processes [21]. Besides solvent extraction, steam distillation and cold pressing are the most employed. The main classes of compounds composing EOs are terpenes, phenols, aldehydes, ketones, and esters. Their amount and composition depend on the plant source and extraction conditions [22].

EOs capture the plant’s natural fragrance and, due to the volatile nature of the compounds at room temperature, they are characterized by a strong aroma. For this reason, they are often used in food products and cosmetics as viable substitutes for synthetic additives, being generally recognized as safe (GRAS) by the U.S. Food and Drug Administration [22,23,24]. Certain essential oils are renowned for their therapeutic and soothing qualities, offering calming effects (such as lavender and chamomile), while others are recognized for their energizing or stimulating benefits (like peppermint and citrus oils) [25,26,27,28].

Saponins are a class of natural chemical compounds found in many plants (ginseng, fenugreek quinoa and other plants from the legume family). Saponins are glycosides, consisting of a water soluble glucidic chain (glycone) linked to a liposoluble structure (aglycone or sapogenin) [29]. Saponins are known for a wide range of biological activities, including antiviral, antifungal, immunostimulatory, and antimicrobial activity. The latter has been widely studied, and it has been observed that saponins extracted from quinoa and camelia seeds have a strong bacteriostatic ability against both Gram−positive and Gram−negative bacteria.

The extraction of these metabolites, typically from fruits and vegetables, provides a sustainable method for imparting targeted bioactive properties to polymer matrices. The integration of these molecules not only adds value to waste-derived compounds, but also improves the performance of biopolymers in applications like food packaging, pharmaceuticals, and medical devices, aligning with circular economy principles.

Avocado fruits and seeds are rich in phytochemicals like polyphenols, triterpenoids, acetogenins, and fatty acids, known for their antihypertensive, antimicrobial, antioxidant, and hypolipidemic benefits. Kupnik et al. successfully recovered biologically active molecules from avocado seeds using various extraction methods [30]. Phenolic compounds with strong antimicrobial activity and antioxidant proteins were employed to enrich bacterial cellulose membranes, showing excellent antibacterial properties and a promising potential for biomedicine, pharmaceutical, and cosmetic applications [31]. Salem et al. carried out a comprehensive analytical and biological evaluation of selected vegetable by-products (potato, onion, and garlic peels), demonstrating the high content of several metabolites, like phenolic acids, flavonoids, saponins, and alkaloids, with promising antimicrobial, anti-osteoarthritis and wound healing potentials [32]. Bioactive extracts obtained from onion and potato peels were used to enhance the antibacterial and antioxidant activity of low-density polyethylene (LDPE) films for food packaging applications [33].

## 3. Extraction Methods of Bioactive Molecules—State of the Art

Extraction is a crucial step in isolating and recovering different bioactive molecules present in agri-food waste biomasses, contributing to their potential reuse in several applications. The choice of extraction and purification methods is crucial not only for maximizing the yield, but also for ensuring the quality and purity of the final extracts. Before extraction, biomass typically undergoes pre-treatment to achieve a uniform, reduced particle size. This step is essential for increasing the contact area between the raw material and the extractant, thereby enhancing their interaction and optimizing extraction efficiency [34,35]. In particular, the biomass is generally dried or lyophilized to completely remove water, and subsequently cut, milled, and sieved. 

Different extraction methods are employed to recover valuable molecules of interest from waste biomasses. Table 1 summarizes the main extraction methods along with their respective advantages and disadvantages. 

These methods can be used either individually or in combination to enhance the extraction yield and improve the purity of the final product. Among them, solvent extraction is one of the most used. It involves using a solvent to selectively dissolve and remove one or more components from a mixture, based on differences in solubility or affinity between the substances. The selection of the solvent plays a key role and takes into account different factors such as the polarity of the compound to be extracted, the nature of the matrix, and the desired purity. Temperature, time, and the type of solvent are the most important factors affecting the yield and the purity of extracted components in this process. The most commonly used techniques include solid–liquid extraction methods such as Soxhlet extraction and maceration.

The Soxhlet extraction (SE) utilizes a device capable of separating, from a solid mixture, components which are poorly soluble from those which are insoluble using a volatile solvent, in a continuous manner. A Soxhlet apparatus consists of three main parts: a solvent-containing flask, an extractor, and a water-cooled reflux condenser. The solid material, placed in a porous cellulose thimble inside the extractor, is repeatedly washed by solvent vapors that condense and drip back into the chamber. When the chamber fills, the siphon automatically empties it, allowing the solvent, now enriched with the extracted compounds, to return to the flask. This cycle repeats for hours or days, concentrating the extracted substances in the flask. The main disadvantages of SE include long extraction times, which can cause thermal degradation of sensitive materials, the high volume of solvent required, and the absence of agitation to speed up the process.

Maceration (ME) involves immersing finely ground biomass in a solvent mixture, inside a closed container and protected from light, for extended periods, allowing the compounds to diffuse into the liquid. This process results in a fluid extract known as the macerate. ME is commonly employed to obtain bioactive polar compounds from vegetable matrices. Agitation may be applied to increase extraction efficiency, depending on the biomass type. This means that maceration extraction is an energy-efficient method with a lower carbon footprint compared to other extraction methods. There are different types of maceration that change depending on the process specifications: digestion, infusion, and percolation. The digestion is carried out at temperatures between 35 and 65 °C, and in this case, a fluid extract is also obtained. During infusion, the solvent, boiling water, is poured over the crushed biomass, whereas in the percolation, the solvent, by dropping or under pressure, passes through a layer, usually homogeneous, of powdered biomass. 

In recent years, several extraction methods have been studied and applied to replace the traditional methods to enhance efficiency, reduce the use of harmful solvents, lower energy consumption, and increase the yield and purity of bioactive compounds.

Microwave-assisted extraction (MAE) is based on the physical mechanisms of ionic polarization and the reorientation of molecules. In the presence of an electric field, generated by microwave radiation, the polar molecules within the biomass begin to vibrate, so that the dipoles are continuously aligned with the electric field, generate heat which helps break down cell walls, and release bioactive compounds [36]. This technique exhibits several advantages with respect to traditional procedures, including the fine control of pression and temperature, the extremely short extraction times and, moreover, the extraction yields comparable to or higher than those obtained with conventional methods. In addition, this methodology is particularly promising since it is suitable for industrial-scale application [37].

Ultrasound-assisted extraction (UAE) is another technique widely employed to isolate bioactive compounds and other components from different biomasses. Sonication facilitates a complete extraction with higher yields in a very short extraction time. UAE is based on the principle of acoustic or ultrasonic cavitation. The extraction is obtained when high-power and low-frequency ultrasonic waves are coupled into a mixture consisting of botanical material in a solvent. High-power ultrasonic waves are coupled via a probe-type ultrasonic processor in the slurry, and highly energetic ultrasonic waves cross the liquid creating alternating cycles of high pressure/low pressure, which results in the phenomenon of acoustic cavitation [36]. When cavitation bubbles implode on a solid’s surface (starting biomass), microjet and interparticle collisions generate effects such as surface peeling, erosion, particle breakage, sonoporation, and cell destruction, which also facilitate the penetration of solvent into the cell and improve the mass transfer between cell and solvent so that intercellular materials are transferred into the solvent [38]. 

Supercritical fluid extraction (SFE) is an advanced separation technique that uses supercritical fluids (substances at temperature and pressure above their critical point) as a solvent for the recovery of desired compounds from a biomass. This method is particularly valued for its ability to selectively extract compounds with minimal solvent residue and reduced environmental impact. Commonly, carbon dioxide is used for the extraction of non-polar compounds from biomass, due to its non-toxicity, cost-effectiveness, and tunable properties, allowing for efficient extraction. SFE is known for its speed, effectiveness, and the ability to operate at lower temperatures, making it suitable for heat-sensitive materials. Furthermore, this extraction method is highly selective, since supercritical carbon dioxide is fed into the closed system at temperature and pressure which allows the evaporation of the single active ingredient, thus already pure and immediately usable. Three main types of extract can be obtained by SFE, each with unique characteristics. The fluid extract contains active compounds dissolved in the supercritical solvent, but is not ideal for industrial processing and is susceptible to bacterial contamination. The extract is obtained by removing a large amount of solvent, resulting in a solid or semi-solid that is easier to handle and has a lower risk of contamination. Finally, the dry extract is achieved by completely evaporating the solvent, yielding pure active principles that are highly concentrated and free from residual solvents. This type of extract is particularly valuable when purity is essential.

In addition to supercritical extraction, there is subcritical water extraction (SWE), which involves heating water to high temperatures and maintaining it under high pressure. The main advantages of this technique include shorter extraction times, reduced solvent use, and the production of more chemically diverse extracts. A key characteristic of SWE is that as the temperature increases, its polarity decreases significantly, enabling it to behave similarly to organic solvents like methanol or ethanol. This makes subcritical water a more eco-friendly option for extracting a wide range of organic compounds [39].

Pulsed electric field-assisted extraction (PEF) is a promising new technique for the recovery of valuable compounds from waste materials. PEF uses an electric field to create irreversible poration in the cell membrane, increasing membrane permeability [40]. A PEF processing unit consists of several components, including a pulse generator, a treatment chamber, and monitoring devices. The pulse generator is important to generate high-voltage electrical impulses with a specific intensity and magnitude. These pulses are managed by several electrodes distributed in the treatment chamber where the biomass is located. Teflon is generally used as isolating material. Also, the choice of the solvent is crucial to extraction efficiency; in fact, the right solvent could improve electroporation of cell membranes and extraction process. The PEF technique uses electrical waves with a high voltage amplitude. Any product placed in the chamber is subjected to short electrical pulses. Depending on the type of product treated by PEF (i.e., solid, liquid), treatment chambers can be classified into two distinct categories: batch treatment chambers and continuous treatment chambers. The static processing chamber is ideal for processing solid foods. PEF technology efficiently optimizes the extraction of valuable compounds in a shorter time, minimizing solvent and energy consumption at room temperature [41]. The efficiency of the PEF process is influenced by several factors, such as the size of the target component and its location within the cytoplasm or vacuoles, the characteristics of the solvent, and the composition and properties of the employed biomass. 

**Table 1:** Extraction methods commonly used for the extraction of bioactive molecules from agri-food waste.

Extraction Method	Advantages	Disadvantages	Extraction Principle	Ref.
Soxhlet (SE)	High extraction efficiency due to continuous and repeated solvent washing	Long extraction times, high volume of solvent, absence of agitation	Solid–liquid extraction	[34]
Maceration (ME)	Energy-efficient method; lower carbon footprint	Use of organic solvents	Solid–liquid extraction	[35]
Microwave-assisted extraction (MAE)	Pression and temperature control; short extraction times; suitable for industrial scale	Overheating phenomenon	Microwave	[36,37]
Ultrasound-assisted extraction (UAE)	Higher yields; Short extraction times;	High costs; Undesirable molecular changes	Acoustic cavitation	[38]
Supercritical fluid extraction (SFE)	Selectively extract compounds; minimal solvent residue; lower temperatures	Susceptible to bacterial contamination	Supercritical Fluids	[39]
Subcritical water extraction (SWE)	Shorter extraction times; reduced solvent use; more eco-friendly	Susceptible to bacterial contamination	Supercritical Water	[39]
Pulsed electric field-assisted extraction (PEF)	Shorter extraction time; work at room temperature; minimum solvent use	High energy demand	Electrical waves	[41]
Enzyme-assisted extraction (EAE)	Selectively break down cell walls	Not specific final extract	Single or mixture enzymes	[42]
Extractive distillation, hydrodistillation (ED/HD)	Industrial scale; No organic solvents	High temperature	Distillation	[43]

Enzyme-assisted extraction (EAE) is a method used to recover bioactive compounds by using enzymes in addition to the solvent, typically water, rather than organic solvents. EAE exploits the ability of enzymes to selectively break down cell walls, enabling the efficient extraction of active ingredients. Depending on the type of biomass and the specific compounds being targeted, either a single enzyme or a mixture of enzymes may be used. Common enzymes involved in EAE include cellulose and pectinase. The optimization of the EAE focuses on several key parameters: pH, temperature, time, enzyme concentration, and solvent/biomass ratio. The pre-treatment of materials with enzymes, including cellulase, hemicellulose, pectinase, and protease, before extraction aids in cell wall cleavage, significantly increasing the release of molecules incorporated in the starting biomass [42].

Extractive distillation (ED) is a technique widely used at industrial scale [43]. The distiller is a closed system composed of three different sections. The first component consists of a chamber in which biomass is inserted with the solvent, and heated until evaporation. Thanks to the chemical affinity, the solvent takes away with itself the active ingredient by extracting it. The second section is a condensation zone at lower temperatures. The lowering of the temperature causes a reduction in the energy of the system and the evaporated active ingredients pass from the aerial state to the liquid state. A third section, called distillate, serves as fluid collection. An example of distillation is steam distillation, where water acts as the solvent. In this process, the temperature and pressure of the steam are the key variables to enhance the separation efficiency. ED is performed by adding a high-boiling solvent to the biomass, which serves as a separating agent. This solvent increases the relative volatility of the components, making it easier to separate them during distillation. Another similar extraction technique is hydrodistillation (HD), which represents a variant of steam distillation. This methodology is mainly used to recover essential oils and bioactive compounds without using organic solvents. HD involves different physicochemical processes such as hydrodiffusion, hydrolysis, and decomposition by heat. For HD extraction, the biomass is soaked for some time in water; after this period, the mixture is heated and the volatile materials are transported by steam, condensed, and separated. 

## 4. Bioactive Molecules Extracted from Agri-Food Wastes with Antimicrobial Properties: Recent Advances

### 4.1. Phenolic Compounds

Fruit and vegetable peels are one of the major components of agri-food waste. They are generally richer in phenolic compounds than the edible portion of the fruit; therefore, the recovery and valorization of this waste can be particularly interesting [44]. There are numerous recent studies that have evaluated the possibility of extracting polyphenols from fruit and vegetable peel waste and have tested their antimicrobial properties. Some examples of these studies are listed in Table 2.

#### 4.1.1. Phenolic Compounds from Fruit Wastes

Citrus peel waste has been widely investigated as a potential resource of polyphenolic compounds [45]. Recent investigations have focused on various fruits, including pomegranate [46,47], mandarin [10], pineapple [38], and orange [48]. Kupnik et al. [46] explored different methods to extract biomolecules from pomegranate peels. They compared conventional extraction methods like ME, UAE, and SE with SFE. ME and SE facilitated the extraction of higher amounts of total polyphenols with respect to the UAE and SFE (i.e., approx. 40 mg Gallic Acid Equivalent (GAE)/g obtained for both ME and SE, approx. 25 mg GAE/g observed for UAE and SFE). Pomegranate peel extracts (PPEs) were particularly rich in catechins, and ellagic and gallic acid, and showed strong antimicrobial activity both against Gram+ and Gram− bacteria strains. The disc diffusion method (DDM) [49] and broth microdilution method (BMM) were taken in consideration. In particular, the microbial growth inhibition rates (MGIR) of *E. coli*, *P. fluorescens*, *P. aeruginosa*, *B. Creus*, and *S. aureus* were higher than 95% when 2.7 mg/mL of pomegranate extracts by SFE were used. PPE showed antibacterial activity also when encapsulated in a gel, as reported by Rifna et al. [47]. They explored ionic gelation (by sodium alginate and calcium chloride) as an encapsulation method to stabilize total hydrolysable tannins (THT) such as gallic, tannic, and ellagic acid, present in higher quantities in PPE. The pomegranate extract employed for the encapsulation was obtained by UAE using acetone as solvent. An encapsulation efficiency of 83.7% was obtained when 40 g/kg of sodium alginate, 25 g/kg of calcium chloride and 300 g/kg of pomegranate peel extract were used. This formulation was highly active against *S. aureus* with minimum inhibitory concentration (MIC) of 0.3 mg/mL (agar well diffusion method). 

Mandarin peel extracts (MPEs), rich in flavanone, were obtained by MAE using a methanol/dimethyl sulfoxide mixture as solvent [10]. MPE was then used to form bioelastomers using polydimethylsiloxane (PDMS) in different ratios. The bioelastomer containing 15 wt.% of MPE (B-MPE15%) exhibited antimicrobial activity (agar well diffusion test) against both *S. aureus* and *E. coli*, with a higher performance against the Gram+ *S. aureus*. B-MPE15% had an estimated amount of mandarin extract of 53.7 mg per 50 g of the bioelastomer. 

Exotic fruits, such as tropical fruits, have been extensively studied for their phytochemical properties [50]. In particular, their very high content of polyphenols is well known and particularly promising [51]. Numerous studies were conducted on the antimicrobial properties of extracts from the peels of exotic fruits. Recently, Koirala et al. [52] compared the phytochemical composition of mango and radish peel extracts (by maceration in 80% ethanol solution). The total phenolic content (TPC) was significantly higher in the extract from mango peels (280 μg quercetin/g of extract powder) than radish peels (26 μg quercetin/g of extract powder). A broth microdilution assay revealed that mango peel extract exhibits antimicrobial activity against both Gram+ (*B. subtills* and *S. aureus*) and Gram− (*E. coli*, *S. typhimurium*) bacteria strains with a minimum inhibitory concentration (MIC) of 0.125 mg/mL, whereas radish peel extract always showed a MIC higher than 2.5 mg/mL. 

Similarly, Budiati et al. [53] studied several tropical fruit peels, stems, and leaves as source of active biomolecules (pineapple, jenggkol, bitter bean, jackfruit, durian, coffee, mangosteen, sembukan, lamtoro, cacao, and Indian marsh fleabane). Extractions were conducted by maceration in 96% methanol at RT. Coffee, mangosteen, jengkol, and bitter bean extract showed the highest TPC (4.8 mgGAE/g for the coffee extract, 4.4 mgGAE/g for the others). However, they found that extract from mangosteen peels (but also jengkol and bitter bean extract) presented higher antimicrobial activity (disc diffusion assay) than coffee extract against different Gram+ (*B. cereus*, *L. monocytogenes* and *S. aureus*) and Gram− (*E. coli*, *S. Typhimurium*, *V. parahaemolyticus* and *A. hydrophilla*), probably due to the presence of some type of polyphenols (mainly apigenin, myricetin, catechin, quercetin and gallic acid) not found in the coffee extract. Interestingly, sembukan and lamtoro extract showed greater antimicrobial activity against Gram− bacteria.

Chaiwarit et al. [54] developed an active packaging using extracts from different tropical fruit peels as antimicrobial active additive. Specifically, the peels of three tropical fruits from Thailand (*Garcinia mangostana*, *Nephelium lappaceum*, and *Mangifera indica*) were extracted by both ME and MAE techniques using water, ethanol, and a water/ethanol 40:60 solution. The extracts, rich in phenolic acids and flavonoids, were tested by disc diffusion method against *S. aureus* and *E. coli.* Extract from *Garcinia mangostana* obtained by MAE in a water/ethanol 40:60 solution showed the highest concentration of total polyphenolic compound (coded as MT-MAE-W/E, 144 mg GAE/g). On the contrary, mango peel extract consistently showed the lowest TPC with any extraction method. MT-MAE-W/E present the highest antibacterial activity against both *S. aureus* and *E. coli*. Only this extract demonstrated growth inhibition zones against both *S. aureus* and *E. coli* (9.5 and 9.9 mm of the clear zone at concentration of 100 mg/mL of extracts). For this reason, a hydroxypropyl methylcellulose (HPMC) film containing MT-MAE-W/E was prepared by the solvent casting method. The active film exhibited higher antibacterial activity against *S. aureus* than *E. coli* (i.e., 30.2 and 26.5 mm of growth inhibition zone, respectively).

Active packaging based on the addition of tropical fruit extracts has also been developed by Sganzerla et al. [55]. They added different amount of *Acca Sellowiana* powdered peels (starting from 0.4 to 4 wt.%) to an aqueous starch solution in order to obtain an edible coating with antimicrobial properties.

Antimicrobial properties were evaluated by the disc diffusion assay against three different bacteria strains: *E. coli*, *S. typhimurium*, and *P. aeruginosa*. Edible films containing 1% *Acca Sellowiana* powders were found to weakly inhibit the growth of *E. coli*, with an inhibition zone from 5 to 7 mm. Increasing the amount of powders from 1% to 2%, the film was also able to weakly inhibit the growth of *S. typhimurium*. Interestingly, they found that the addition of only 3 wt.% of *Acca Sellowiana* powder into the film resulted in a response against the three different bacterial strains (*E. coli*, *S. typhimurium*, and *P. aeruginosa)* with an inhibition zone between 8 and 10 mm for *E. coli* and *S. typhimurium*, and from 5 to 7 mm for *P. aeruginosa*.

**Figure 2:**
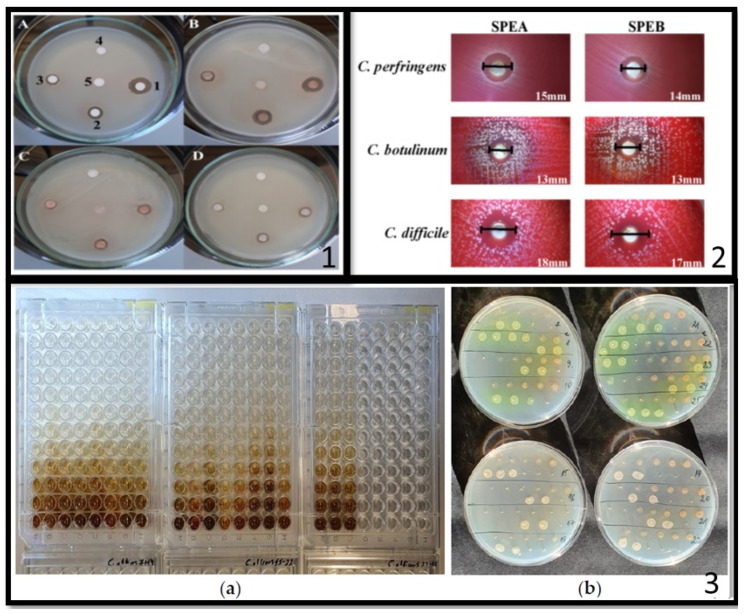
Some examples of antimicrobial tests carried out using biomolecules obtained from agri-food waste valorization. (**1**) Antimicrobial activity of four grape seed extracts (**A**–**D**) against *S. aureus* (zone 1, 0.50 mg/mL extract; zone 2, 0.25 mg/mL; zone 3, 0.10 mg/mL; zone 4, 0.05 mg/mL; zone 5, negative control) (reproduced from [60]). (**2**) Inhibition halos obtained for *C. perfringens*, *C. botulinum* and *C. difficile* in the screening test using two different extracts of saffron petals (SPE A and SPE B) (reproduced from [70]) and (**3**) (**a**) MIC of ‘Maria Bruvele’ extract against *C. albicans* by the two-fold serial broth microdilution method; (**b**) MBC of the same extract against *P. aeruginosa* and *C. albicans* (reproduced from [65]).

Seeds [56] and leaves [57] of fruits also contain higher concentrations of polyphenols than the edible part of the fruit. For this reason, several researchers have evaluated seed and leaf wastes as a potential source of biomolecules with potential antimicrobial activity. As an example, different extraction methods were explored by Kupnik et al. [30] on avocado seeds. They tested UAE (using water as solvent), SE (with ethanol as solvent), and CO_2_ SFE. They observed that UAE allows the extraction of the highest amount of some valuable polyphenolic compounds (as epicatechin, hexperidin, quercetin, gallic acid, 2,3-dihydroxybenzoic acid, cinnamic acid), whereas SFE showed the worst performance in the extraction of these polyphenols (i.e., UAE: 394 mg/100 g; SE: 351 mg/100 g; SFE: 178 mg/100 g). However, the latter was found to be the only one capable of extracting quercetin from avocado seeds. The different polyphenol composition of the three extracts led to an interesting behavior of the extracts against bacterial strains. Specifically, the UAE extract was found to be richer in phenolic acids and presented a greater antimicrobial activity against Gram− bacteria. On the contrary, the extract obtained by SE, with a higher concentration of flavonoids (in particular, hesperidin), showed a better activity towards Gram+. The UAE and SE extracts were not particularly active in inhibiting fungal proliferation, whereas the SFE extract was notably effective against fungal growth, confirming a possible key role of quercetin in inhibiting fungal growth [58]. Sirichan et al. [59] explored the antimicrobial potential of makiang seed, extracted by the UAE technique using 70 wt.% ethanol solution. They studied the effect of temperature, time, and amplitude on the chemical composition of the extracts and found the best condition to be as follows: temperature of 52 °C, time of 32 min, and amplitude of 40.5%. The extracts were rich in gallic acid and showed a MIC of 1.56 mg/mL against *S. aureus*, *L. plantarum* and *S. cerevisiae* and 6.25 mg/mL against *E. coli*. Krasteva et al. [60] studied grape seed extracts of four different grape varieties (*Pinot Noir*, *Marselan*, *Cabernet Sauvignon*, and *Tamyanka*). They conducted the extraction by maceration in a 70% ethanol solution at room temperature for 5 h and they found that grape seed extracts are rich in catechin (flavonoids) more than phenolic acids. As in the results of Kupnik et al. [30], grape seeds extracts, richer in flavonoids (i.e., catechin and epicatechin), exhibited a higher antimicrobial activity against the Gram+ (*S. aureus*) with the respect to Gram− (*E. coli*). In particular, Pinot Noir extracts showed the lowest MIC against *S. aureus* (0.12 mg/mL) and *B. cereus* (0.25 mg/mL). These antimicrobial tests are reproduced in Figure 2. Sánchez-Gutiérrez et al. [61] investigated Olea europaea leaves as resource of bioactive molecules. They explored two extraction methods: Soxhlet and microwave-assisted extraction, using different solvents (water, ethanol, and glycerol). MAE, carried out for 10 min at 80 °C, allowed a higher TPC to be obtained than the respective SE extract, using the same solvent. As an example, the MAE water extract showed a TPC of 19 mg/g DW, whereas the SE water extract presented a TPC of 14 mg/g DW. Although comparable results in the antioxidant properties were obtained for the different extraction methods, the microwave-assisted aqueous extract showed higher antimicrobial activity against *S. aureus* and *Y. enterocolitica* (MIC: 2.5 and 5.0 mg/mL, respectively) than the other extracts (broth microdilution assay). Olive leaves were also investigated by Martiny et al. [62]. The aim of their work was the optimization of the MAE technique. They found the best conditions for the extraction (i.e., the condition that allowed the highest amount of TPC to be obtained) by conducting the process at 100 °C for 2 min at pH6. This extract was found to effectively inhibit *E. coli* at MIC 50 mg/mL.

Several studies were focused on the valorization of other types of plant wastes, such as the external shells of some types of walnuts and hazelnuts. Although they are mainly composed of lignocellulose, they could contain bioactive substances with potential antimicrobial activity. Frazzini et al. [63] investigated the chemical composition of the extracts of cuticle and shell of hazelnuts. The extraction was conducted by UAE, using two different mixtures of solvents (water/acetone 40/60 and water/methanol 50/50) for 2 h at RT. Antimicrobial activity was evaluated against *E. coli*, by broth microdilution method. The polyphenolic profile of cuticle extracts resulted qualitatively and quantitatively higher than that obtained from the fraction consisting of hazelnut shells. Furthermore, the water/acetone 40/60 mixture was more efficient in the extraction of polyphenols. Although both cuticle and shell extracts revealed an antimicrobial effect against *E. coli*, the cuticle extract showed a significantly higher antimicrobial activity than that observed for the shell extract. For example, the MIC observed for the extract in water/methanol 50/50 was 1 and 5 mg/mL, respectively. Arslan et al. [64] investigated the antimicrobial activity of the walnut green husk extract (WGH). Water, methanol, ethanol, acetone, and *n*-hexane were explored as extraction solvents using different temperatures and at different extraction times. The maximum content of total phenolic compounds was found using water at 75 °C for 5 h. Afterwards, they concentrated the extracts by a pressure-driven membrane process using four different membranes (microfiltration, ultrafiltration, nanofiltration, reverse osmosis). Nanofiltration (NF) allowed the highest concentration of total phenolic compounds to be obtained (i.e., 8155 mg/L). The antimicrobial activity of the extract, filtered with different membranes, was evaluated on different bacterial strains: *E. coli*, *P. aeruginosa*, *L. pneumophila*, *E. hirae*, *E. fecalis*, *S. aureus*, *C. parapisilosis*, *C. tropicalis.* WGH resulted active against all the bacteria strain even before the concentration process. However, all the concentrated samples showed higher antimicrobial activity against the bacterial strains tested. In particular, the NF samples showed the highest antimicrobial activity. The bacterial strains most susceptible to WGH were *E. hirae* and *C. parapisilosis*.

Andersone et al. [65] focused on the role of proanthocyanidins (PACs) in the antibacterial and anti-inflammatory response of lignocellulosic waste extracts. Specifically, they explored sea buckthorn (SBT) as waste biomass. Twigs of SBT were ground and macerated at 60 °C in two different solvents: water and water/ethanol 50/50. The extracts obtained in water/ethanol showed a higher concentration of PACs. The purification of PACs from non-tannin and sugar was carried out using 96% EtOH and 70% acetone as purification solvents in a solvent resistant column. The antimicrobial activity of the extracts and that of the purified PACs was tested by broth microdilution method against the Gram+ (*S. aureus* and *B. Cereus)*, the Gram− (*P. aeruginosa* and *E. coli*) bacteria and fungus (*Candida albicans)* (Figure 2). The extracts obtained from the water/ethanol solution showed higher antibacterial and antifungal activity with respect to the water extracts against both Gram+ and Gram− bacteria (i.e., 0.2 mg/mL of MIC against *E. coli* and *S. aureus*). After purification, PACs showed antimicrobial and antifungal activity 4 to 10 times greater than that observed for untreated extracts. The observed results suggested a key role of PACs in the antimicrobial and antifungal properties of these extracts.

#### 4.1.2. Phenolic Compounds from Vegetable Wastes

Salem et al. [32] investigated the phytochemical composition and the potential antibacterial activity of biomolecules extracted by vegetable waste, mostly constituted by potato, onion, and garlic peels. Dried powdered peels were extracted using cold methanol (80 wt.% in water) with the aim of an ultrasonic bath (30 min). By HPLC, 47 different compounds were identified, mostly belonging to the class of polyphenols (i.e., phenolic acids and flavonoids), but saponins and some alkaloids were also found. The extracts obtained from onion peels were found to be the richest in polyphenols and they demonstrated the greatest antimicrobial activity against different bacterial strains, both Gram+ (*S. aureus* and *A. baumannii*) and Gram− (*E. coli* and *P. aeruginosa*). In particular, onion peel extracts showed major antibacterial activity against Gram+ (minimum bactericidal concentration MBC: 3–4 mg/mL for *S. aureus*) with respect the Gram− strains (MBC: 12.5 mg/mL for *E. coli*). Potato peel extract showed activity only against Gram− (MBC: 12.5 mg/mL for *E. coli*; 6.25 mg/mL for *P. aeuriginosa*).

Recently, Jokovic et al. [66] explored the antimicrobial activity of onion peel extracts. Dried onion peels were powdered and suspended in four different solvents: ethyl acetate, acetone, ethanol and methanol for 7 days at room temperature. The acetone extract showed the highest content of TPC and total flavonoids content, corresponding to 60.6 mgGAE/g and 32 mgQU/g, respectively, whereas the ethyl acetate extract showed the lowest TPC (i.e., 25 mg GAE/G). The obtained extracts were tested by microdilution well methods against several bacterial strains (most of which originated from the human gastrointestinal tract). The extracts showed a different activity depending on the employed solvent. The ethanol extract exhibited the highest antimicrobial potential against *P. mirabilis* and *P. aeruginosa* (MIC: 0.78 and 1.56 mg/mL, respectively), whereas, surprisingly, the acetone extract showed the worst antimicrobial activity against the tested microbes. 

Potato peel extracts were also explored by El-Sawi et al. [67]. The extracts, obtained by ME in ethanol (2 days at room temperature), were tested against Gram+ (*S. aureus*) and Gram− (*P. aeruginosa*) strains using agar well diffusion method. The extracts obtained from potato peels proved to be more active against Gram− (10.5 mm of inhibition zone against *P. aeruginosa*) than against Gram+ bacteria (8.0 mm of inhibition zone against *S. aureus*), confirming the results obtained by Salem et al. [21].

Saffron *(Crocus Sativus)* flowers are widely recognized for their phytochemical properties. Used since ancient times in the herbal field, they possess remarkable antioxidant and antibacterial properties since their stigmas and stamens are rich in polyphenols (in particular flavonoids and phenolic acids) [68]. Saffron is also commonly used as a spice in many dishes and its use in the food field generates a potential waste that can be valorized as a source of bioactive molecules with antibacterial properties. Lachguer et al. [69] explored different extraction methods and solvents on saffron flowers waste, collected in Morocco. The first method consists of the decoction of saffron flowers in distilled water at 100 °C for 1 h; the second method consists of the maceration of saffron flower powders in methanol for 48 h; the third method is represented by the extraction with a Soxhlet apparatus by a liquid–liquid hydro-ethanolic solution (6 h). The aqueous solution that results from these processes is extracted with different solvents: diethyl ether, petroleum ether, ethyl acetate, and *n*-butanol. Diethyl ether extract showed the highest total polyphenols and flavonoid content (214 mgGAE/g and 84 mgQE/G), followed by *n*-butanol (172 mgGAE/g and 61 mgQE/G) and ethyl acetate (154 mg GAE/g and 47 mg QE/G). Diethyl ether extract exhibited the largest inhibition zone (16.13 mm) against *S. aureus* at 300 mg/mL. They also evaluated MIC and MBC of the diethyl ether (DE) and ethyl acetate (EA) extract. DE showed the best performance against *S. aureus* (MIC: 12.5 mg/mL; MBC: 25 mg/mL), whereas both the extracts revealed the same antibacterial activity against *E. coli* and *K. pneumoniae* (MIC: 25 mg/mL; MBC: 50 mg/mL). Primavilla et al. [70] tested the antimicrobial activity of two different extracts of saffron petals, extracted by maceration at 45 °C (SPEA) and ultrasonic bath for 10 min (SPEB) using 70% ethanolic solution. SPEA and SPEB showed the presence of polyphenol acids (mainly gallic and chlorogenic acid) more than flavonoid. The SPEA extract showed a higher amount of gallic acid (34.84%) than the SPEB extract (22.22%), in which, however, a higher amount of chlorogenic acid was found (i.e., 46.81% and 32.36%, respectively). Quercetin and kaempferol are the flavonoids mostly found in the extracts. The antibacterial activity of the extract was determined by the agar well-diffusion method. SPEA and SPEB showed antimicrobial activity against *Clostridia perfringens*, *C. botulinum*, and *C. difficile* at a MIB/MBC value ranging between 250 and 500 mg/mL, with the extract SPEA most active with respect to the extract SPEB (Figure 2).

**Table 2:** Recent studies on the valorization of agri-food wastes as resource of phenolic compounds with antimicrobial activity.

Waste	Antimicrobial Molecules	Extraction Method	Potential Application	Ref.
Pomegranate peels	Polyphenols	Different extractions methods: CM, UE, SE, and SFE	Undefined	[46]
Pomegranate peels	Polyphenols, tannins	Pulsed ultrasound-assisted extraction in acetone	Food and pharmaceuticals	[47]
Mandarin peel	Flavonoids	Microwave-assisted extraction	Bioelastomer	[10]
Pineapple	Polyphenols	Ultrasound-assisted extraction	Undefined	[36]
Orange peels and olive leaves	Polyphenols	Ultrasound-assisted extraction	Biomedical application	[48]
Radish and Mango and radish peel	Polyphenols	Maceration in 80% ethanol for 2 h at 40 °C	Undefined	[52]
Peels and seeds of tropical fruit	Flavonoids	Maceration in methanol at RT	Industrial use	[53]
Peels and seeds of different tropical fruit	Polyphenols flavonoids	Maceration and MAE in water, ethanol 95% and water–ethanol	Active Food packaging	[54]
Feijoa Peel Flour (FPF) from *Acca sellowiana*	Polyphenols, flavonoid	No extraction: drying and homogenization of the peels	Edible coating	[55]
Avocado seeds	Flavonoids, quercetin, anthocyanidins	UAE using water as solvent SE using ethanol as solvent Supercritical carbon dioxide	Medicine, pharmaceutical, cosmetic	[28]
Makiang seed	Polyphenols, flavonoids	Ultrasound-assisted extraction in 70% ethanol solution	Extended shelf-life of food	[59]
Seeds from four grape variety (Cabernet, Marselan, Pinot Noir, and Tamyanka)	Flavonoids, anthocyanin, procyanidin	Maceration in 70% ethanol	Antimicrobial agents in food products	[60]
*Olea europaea* L. leaves	Flavonoids, Cinnamic acid	SE and MAE with different solvents: distilled water, ethanolic and glycerol	Undefined	[61]
*Olea europaea* L. leaves	Polyphenols	Microwave-assisted extraction	Natural additives	[62]
Hazelnut cuticle and shell	Polyphenols	Maceration in water/EtOH 50:50 and acetone/water 40:70	Undefined	[63]
Walnut green husk (WGH)	Polyphenols	Maceration in 5 solvents: *n*-hexane, acetone, ethanol, methanol, water	Undefined	[64]
Lignocellulosic waste	Oligomeric proanthocyanidin	Convective extraction at 60 °C (30 min) in water or water/ethanol (1:1)	Antibacterial and antifungal	[65]
Onion, potato and garlic peel	Polyphenols, flavonoid	UAE with cold 80% methanol (30 min)	Undefined	[30]
Onion peel	Polyphenols	Maceration by four organic solvents: methanol, ethanol, acetone, and ethyl acetate	Potential pharmaceutical application	[66]
Potato and taro peels; Husk and silk of corn.	Polyphenols, flavonoid	Maceration in 70% ethanol	Therapeutic use	[67]
Saffron flowers	Polyphenols, flavonoids	Decoction in water for 1 h at 100 °C. Maceration in methanol for 48 h. SE by liquid–liquid hydro-ethanolic solution for 6 h	Food system as a natural preservative	[69]
Saffron flowers	Polyphenolic acid and flavonoids	SPE A: maceration in ethanol 70% at 45 °C. SPE B: UAE in Ethanol 70% for 10 min at RT	Food industry	[70]

### 4.2. Essential Oils

Several studies have demonstrated that essential oils possess antibacterial, antifungal, and anti-parasitic properties [71,72,73,74,75,76], which has led to a significant increase in their usage (Table 2). 

Among the various parts of plants that are used to extract EOs, waste leaves and fruit peel are particularly rich in these components and widely reported the literature. Petretto et al. [77] investigated the cytotoxicity, antimicrobial, and antioxidant activity of EOs isolated by hydrodistillation from discarded leaves of citrus limon plants. EOs exhibited DPPH radical scavenging ability and more selective toxicity towards cancer cells than normal cells. The antimicrobial activity was tested against 8 bacterial and 2 yeast species through the culture broth microdilution method and measuring the minimum inhibitory concentrations (MICs) after 24 h of incubation. It was found that the Gram+ bacteria were more susceptible to EOs than Gram−. In particular, a better inhibitory activity was found against *L. monocytogenes*, *S. aureus* (MIC values from 2.5 to 5 μL/mL), and *C. albicans* (MIC = 0.625 μL/mL). 

Similar observations were reported in the extensive study, even if less recent, carried out by Fancello et al. [78]. This investigation assessed the antimicrobial activity against food related microorganisms of EOs obtained from leaves of *Citrus limon* var. *pompia*. EOs were obtained through hydrodistillation and were mainly composed of limonene, geranial, and neral. The in vitro antimicrobial activity was assayed against 12 bacterial and 4 yeast species by the microdilution broth method, according to the ISO 10932 (ISO (International Organization for Standardization), 2010) standard [79] and M07-A9 standard (Clinical and Laboratory Standards Institute, 2014) for lactobacilli and pathogens [80], respectively. After incubation at 37 °C for 24 h, MICs (μL/mL), values were determined as the lowest EO concentration that inhibited the visible growth of the tested microorganism, which was indicated by the absence of turbidity. DMSO alone (at 1%) was used as a negative control. The strain members of *L. genus*, which include some of the most important bacteria in food fermentations, along with the pathogens *S. enterica* and *E. coli*, showed the highest antimicrobial resistance against *pompia* EOs. Conversely, all tested *L. monocytogenes* and *S. aureus* strains exhibited a high sensitivity at a low concentration, in agreement with other works demonstrating that antimicrobial activity was strain-dependent [81,82,83]. In one of those works, the authors also suggested that the high inhibitory activity of their EOs could be due to the high concentration of oxygenated compounds (such as monoterpene) [83], which represented 58.5% of the EO composition. 

Martínez-Abad et al. [84] used lemon peel waste to extract the EOs applying microwave-assisted hydrodistillation (MAHD). EOs were composed by six major compounds as revealed by GC-FID: limonene (65.082 wt.%), β-pinene (14.517 wt.%), γ-terpinene (9.743 wt.%), sabinene (2.395 wt.%), α-pinene (1.992 wt.%), myrcene (1.427 wt.%). Minor components represent amount <1 wt.%. The antimicrobial properties against *E. coli* and *S. aureus* strains, tested with the traditional broth macrodilution method at concentrations of EO ranging from 15 to 5000 ppm showed strong bacteria inhibition. In the measurements, the control samples of both bacteria showed a growth increase of approximately two log units after 24 h of incubation. The initial inoculum sizes were 5.72 ± 0.26 log CFU/mL for S. aureus and 5.80 ± 0.01 log CFU/mL for *E. coli*. EO extracts demonstrated some growth inhibition of *S. aureus* at a concentration of 50 ppm, while bacteriostatic effects were achieved at 150 ppm for *S. aureus* and 500 ppm for *E. coli*. Finally, a 99.9% reduction in the inoculated bacteria was achieved at concentrations exceeding 1500 ppm in both cultures, leading to bacterial counts below 1 for *S. aureus* and approximately 1.45 for *E. coli*.

Grover et al. [85] studied the antimicrobial activity of essential oil from three different galgal cultivars by using hydrodistillation, steam distillation, MAE, UAE, and SFE. They found that among the tested extraction methods, the hydrodistillation demonstrating superior performance compared to other methods. EOs extracted from the fruit peels contained varying amounts of D-limonene, α-ocimene, α-myrcene, and α-pinene were common among all cultivars with difference in composition. D-limonene was found to be the predominant compound in all samples, with concentrations ranging from 43 to 89%. The antimicrobial outcomes indicated that there was a notably (*p* < 0.05) greater zone of inhibition against *S. aureus* in comparison to *E. coli*, according to the previous results.

### 4.3. Other Molecules

This paragraph includes additional examples of molecules with antimicrobial properties extracted from waste materials, which, however, are not extensively covered in the scientific literature (Table 3).

Saponins are found in many edible and non-edible plants, typically as complex mixtures of lipid-soluble glycosides, comprising water-soluble glycones chemically bonded to either triterpenoids or steroids. They exhibit a wide range of biological effects, influenced by their chemical structure and including antimicrobial, anti-cancer, adjuvant, and gastroprotective properties [86,87,88,89]. Zhao et al. [90] extracted tea saponin from industrially produced *Camellia oleifera* shells by using 75% ethanol aqueous solution. High purity (i.e., 82.5%) was obtained by means of a macroporous resin in optimized conditions. Tea saponin demonstrates a potent bacteriostatic effect against *S. aureus* (Gram+) and *E. coli* (Gram−), with MIC of 0.5 and 1 mg/mL, respectively. Its minimum bactericidal concentration is 4 mg/mL. The authors also studied the antibacterial mechanism through the growth, morphology, and extra-cellular proteins of bacteria and found that tea saponin exerted its good bacteriostatic effect through destroying the cell membrane structure and inhibiting the growth of bacteria. 

The literature also reports the interesting antimicrobial activity of water extract mixtures from waste leaves and seeds. Water-based extractions are greener and more sustainable, reducing the need for harmful chemicals and minimizing environmental impact with respect to the extraction with organic solvent which contribute to pollution and require additional energy for solvent recovery. Also, water is inexpensive and readily available, while organic solvents can be costly and require specialized equipment for safe handling, storage, and disposal. Casillo et al. [91] investigated the antibiofilm activity of water-soluble extracts obtained under different pH conditions from *Cannabis sativa* seeds and from previously defatted seeds. The extracts did not show cytotoxicity to HaCaT cells and significantly inhibited the formation of Staphylococcus epidermidis biofilms. From the 2D-NMR analysis, it was highlighted that glycerolipids are probably responsible for the observed antibiofilm effect, but a possible synergistic contribution by other components was not excluded.

**Table 3:** Recent studies on the valorization of agri-food wastes as resource of EOs and other molecules with antimicrobial activity.

Waste	Antimicrobial Molecules	Extraction Method	Potential Application	Ref.
*Citrus limon* leaves	Essential oil rich in citral and limonene	EO isolated by hydrodistillation from the discarded leaves of lemon (*Citrus limon*) plants	Food, cosmetic and pharmaceutical industries	[77]
*Citrus limon* var. *pompia* leaves	Limonene, geranial and neral	EO isolated by hydrodistillation	Cosmetic and pharmaceutical industries	[78]
*Citrus limon* peel	Essential oil rich in limonene	Microwave-assisted hydrodistillation (MAHD) with a Clevenger apparatus	Natural pigments and antimicrobials	[84]
Peel of different galgal (Citrus Pseudolimon) cultivars	Limonene, ocimene, myrcene, and pinene	Various methods: Clevenger apparatus, steam distillation, MAE, UAE, and SFE	Undefined	[85]
*Camelia oleifera* shell	Saponins	Maceration in 75% ethanol	Undefined	[90]
*Cannabis sativa* seeds	Glycerolipids	Maceration in water	Human health	[91]

## 5. Mechanism of Antimicrobial Activity of the Different Class of Bioactive Molecules

Biomolecules extracted from waste biomasses (i.e., polyphenols, essential oils, and saponins) exert their antimicrobial activity through a variety of mechanisms [92,93]. These natural compounds are generally known for their ability to interact and/or damage the cell membranes of microorganisms, altering their permeability up to causing the cell membrane disruption [94]. They can influence metabolic processes or inhibit the synthesis of key components for the growth and development of bacteria such as some proteins or nucleic acids. They can also inhibit the formation of bacterial biofilm [95,96]. 

Moreover, antimicrobial mechanisms strongly depend on the type and chemical structure of the involved molecules as well as on the bacteria species studied [97].

**Figure 3:**
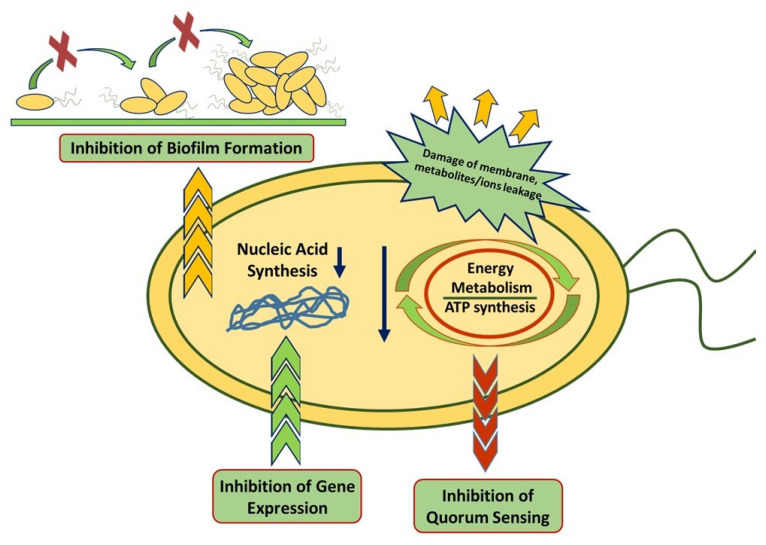
Schematic diagram of antimicrobial mechanisms exerted by biomolecules extracted from waste biomass.

A schematic diagram of the different mechanisms of action of these antimicrobial compounds is reported in Figure 3.

The most common antimicrobial mechanisms of polyphenols can be summarized in three categories:

### 5.1. Alteration of the Cell Membrane

Polyphenols can inhibit the synthesis of the cell wall in bacteria, a key target for many antimicrobial agents. As example, Wu et al. [98] demonstrated that two flavonoids (apigenin and quercetin) are able to inhibit d-Alanine:d-Alanine ligase, an enzyme involved in the assembly of the peptidoglycan precursor. Similarly, fatty acid synthase II (FAS-II) which catalyzes fatty acid chain elongation, is inhibited by the action of different flavonoids such as butein, fisetin, isoliquiritigenin, 2,2′,4′-trihydroxychalcone, and epigallocatechin gallate [99,100]. Polyphenols can also destabilize the cell membrane, acting on its permeability and leading to the leakage of intracellular content, causing its disruption. This effect is often mediated by the interaction that some polyphenols can generate with membrane proteins, such as the efflux pump [101]. Catechins extracted from tea possess the ability to bind to the lipid bilayer of bacterial membranes, inactivating or inhibiting the synthesis of intracellular and extracellular enzymes that ultimately causes membrane disruption and then cell death [102].

### 5.2. Inhibition of Energy Metabolism and the Bacterial DNA Synthesis of Bacteria

Several polyphenols can act as inhibitory substrates for some enzymes involved in microbial metabolism (such as those implicated in cellular respiration), leading to a significant decrease in metabolic activity. A partial inhibition of ATP synthase (in some cases up to 60%) is observed for several types of polyphenols: diosmin, kaempferol, chrysin, hesperidin, and apigenin [103]; genistein and resveratrol [104]; piceatannol, resveratrol, quercetin or quercetin-3-β-D glucoside [105]; cocoa polyphenols [106]; wine grape phenolic extracts [107]. The inhibition of dihydrofolate reductase by some types of catechins leads to a significant reduction in metabolic activity and inhibition of fatty acid and nucleic acid synthesis [108].

### 5.3. Disruption of Quorum Sensing and Biofilm Formation

The ability to form biofilms is an important property of various bacteria species and is associated with quorum sensing (QS, a cell-to-cell communication system) [109]. Biofilm is an assembly of microbial cells that are irreversibly bound to a surface with bacteria embedded in a self-produced matrix of extracellular polymeric substances (EPSs), such as polysaccharides, proteins, and DNA [110]. This matrix constitutes a protective layer that helps to protect the bacteria from environmental stresses, such as antibiotics, immune system attacks, and disinfectants, allowing the bacteria within the biofilm to survive in harsh conditions [111]. The ability of some polyphenols to interfere with QS, thus inhibiting biofilm formation, makes the microbial substrate more susceptible to external agents. Among these, quercetin interferes with the QS of several bacterial species, showing excellent efficacy in inhibiting biofilm formation [112,113,114]. 

The antibacterial activity of polyphenols is strongly influenced by their molecular structure. In particular, the presence and position of hydroxyl groups on the aromatic rings of flavonoids seems to play a key role in their antimicrobial activity [90]. The presence of at least two hydroxyl groups (preferably in position C5 and C7) further increase the antimicrobial activity of flavones [115]. Moreover, the reduction of the hydroxyl group due to intramolecular hydroxyl cyclization, along with the substitution of –OCH_3_, results in a decrease in the antibacterial activity of polyphenols [116]. Although presenting the same degree of lipophilicity and a similar arrangement of oxygenated groups on the molecular structure, planar flavone was found to have a greater antimicrobial activity than a non-planar flavanone, demonstrating that molecular geometry can also play a fundamental role in the antimicrobial activity of these molecules [117].

Finally, it is widely reported in the literature that Gram− bacteria strains are more resistant to phenolic compounds than Gram+ [118]. This is probably related to the presence of an external membrane, with a high phospholipid content, which makes the cell wall impermeable to polar molecules such as polyphenols [119,120]. The resistance of Gram− bacteria strains to phenolic compounds could also be associated with the action of particular enzymes, present in the periplasma, which are able to break down molecules introduced from outside the cellular environment [102,121].

The terpene fraction contained into essential oils is mainly responsible for their antimicrobial activity. Unlike polyphenols, which show different antibacterial mechanisms, terpenes have as their main target the bacterial cell membrane [93]. Several studies suggested that terpenes are able to produce a strong bacteriostatic interaction, binding some membrane proteins or other essential constituents for cell growth [93,122]. They are therefore able to form an external layer to the membrane that modifies the electrostatic potential of the cell and damages the integrity of the membrane, resulting in internal cellular components release [123]. Some authors, however, also reported the effects of essential oils on quorum sensing and the inhibition of biofilm formation [124,125].

**Figure 4:**
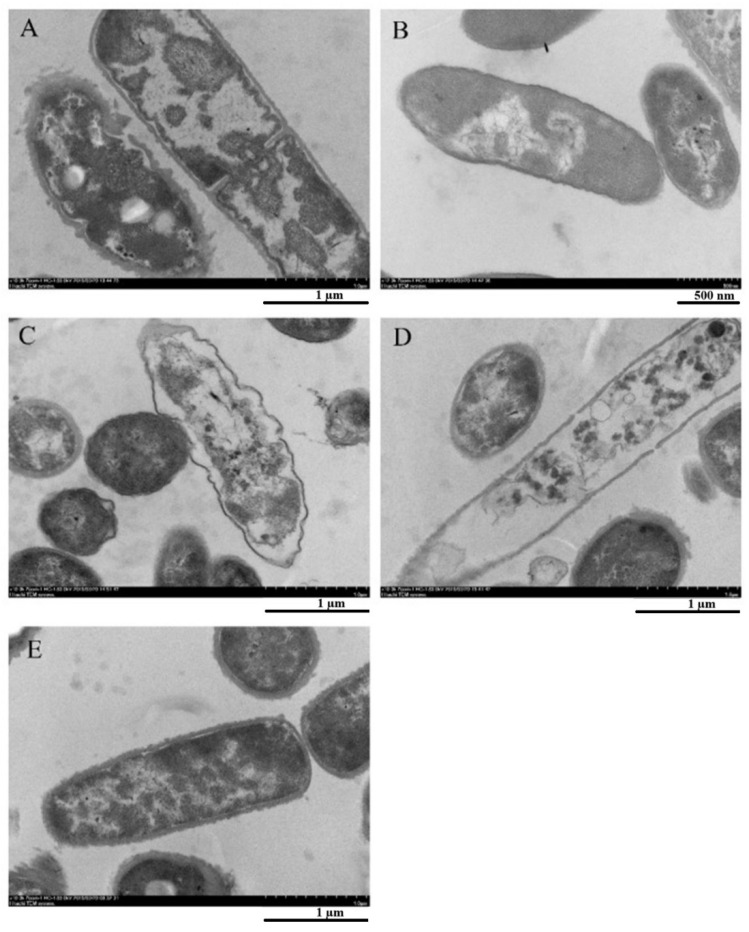
*B. cereus* morphology observed by TEM. (**A**) *B. cereus* treated with 1/2 MIC. (**B**) *B. cereus* treated with MIC. (**C**) *B. cereus* treated with MBC. (**D**) positive control (*Cefixime*). (**E**) negative control (untreated *B. cereus*). Reprinted with permission from Ref. [126]. Copyright 2020 by Elsevier. For more clarity, a scale bar has been added under each image.

The antimicrobial action of saponins, instead, consists of binding to the cell membrane, damaging it, and bringing it to complete rupture [90,126]. Dong et al. [126] showed the effect of saponins extracted from quinoa husk on the morphology of *B. cereus* cell membrane. SEM micrographs, reported in Figure 4, show how saponins cause severe damage to the bacterial cell membrane, including a clear degradation of the cell wall, observable in Figure 4A,B (i.e., treated with minimum inhibition content of saponin) which can be followed by the rupture of the cytoplasmic membrane and membrane proteins when the concentration reaches MBC (Figure 4C). Cefixime has been used as positive control (Figure 4D) whereas untreated strain of *B. cereus* is depicted in Figure 4E.

Also sulfur-containing compounds, such as those found in garlic and onions, can inhibit enzymes vital to bacterial survival, disrupt cell membrane function, and generate oxidative stress [127].

## 6. Potential Applications

**Figure 5:**
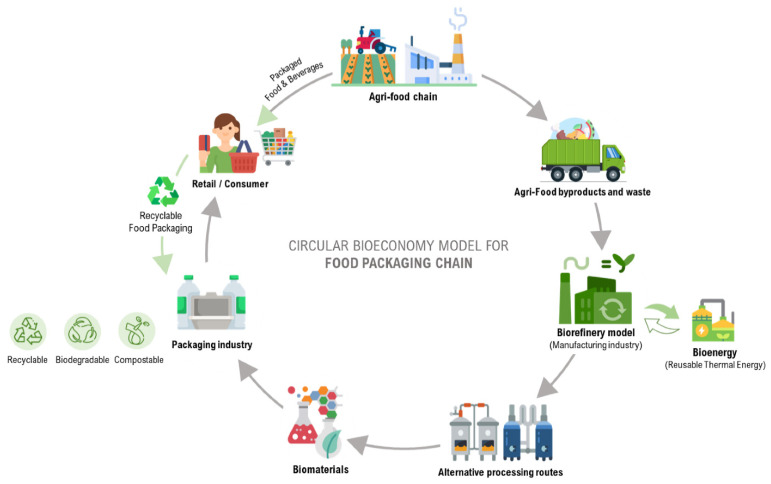
Circular bioeconomy model in food packaging: integrating renewable resources in biorefineries to produce recyclable, eco-friendly materials. Reproduced from [127].

Molecules with antibacterial properties extracted from food industry residues include a wide range of compounds, which have a wide range of applications ranging from cosmetics to food packaging (Figure 5). 

In particular, they can be incorporated into food packaging materials to inhibit microbial growth, thereby enhancing food safety [128]. Different studies have demonstrated the effectiveness and suitability of bioactive metabolites extracted from food wastes in this application field. Rifna et al. [47] extracted from pomegranate peel waste, via pulsed UAE, tannins with potential applications in food and pharmaceuticals. As tannins are subject to reduced stability and performance when applied in food, the strategy was to encapsulate them within a matrix to avoid their degradation, using the external gelation. The probable mode of action of encapsulated pomegranate peel waste was revealed as the depolarization of the bacterial membrane. The results showed that the antibacterial activity of the encapsulated extracts against organism is the same of the synthetic antibiotics used generally to kill microorganisms in food. Encapsulated extracts produced using the external gelation technique at the optimized condition displayed superior storage stability possessing strong antimicrobial activity when compared to encapsulated extracts produced using the spray drying technique. The study assesses the importance of nature-derived compounds to enhance the quality and safety of consumables. Krasteva et al. [60] extracted polyphenols, flavonoids, anthocyanin, procyanidin using 70% aqueous ethanol from four different grape seeds. The aim of the study was to evaluate the total phenolic content, composition, and antioxidant and antibacterial activities of this biomass. The antimicrobial activity of the obtained extracts was also evaluated against *S. aureus*, *B. cereus*, and *E. coli* using the agar diffusion test and a test to determine the minimum inhibitory concentration (MIC). The extracts show a good potential as antioxidant and antibacterial agent. The high antioxidant and antimicrobial activity of the extracts allow their application as antimicrobial agents in food, reducing the risk of allergic reactions given by synthetic preservatives. Chaiwarit et al. [54] performed the extraction, through MAE and maceration with water, of antibacterial molecules from tropical fruit peels and formation of HDMC film containing this extract in order to develop an antibacterial packaging material. Rude extracts of mangosteen, rambutan, and mango peels exhibited different percentage yields, flavonoids, phenolics, and antibacterial properties. Furthermore, different extraction methods and solvents influenced these characteristics. The study demonstrated that tropical fruit extracts could be an alternative additive used for antibacterial active film packaging. The findings from the study may be useful for further studies to purify the extract in order to use the natural active compound in film formulation for active antibacterial packaging. Sganzerla et al. [55] developed an edible coating with antimicrobial properties adding to an aqueous starch solution different amount (starting from 0.4 to 4 wt.%) of *Acca Sellowiana* powdered peels as an antimicrobial agent.

Another exploitation of secondary metabolites extracted from agri-food wastes, in the food sector, is their incorporation into formulations of natural preservatives, with the purpose to enhance the shelf life and safety of food products. Lachguer et al. [69] evaluated the primary phytochemical content, the antioxidant properties, and the antimicrobial activities of different extracts of saffron flower wastes and against bacterial and fungal strains involved in diverse pathologies. This multifaceted study revealed that saffron flower extracts were a potential natural antioxidant source and optimal antimicrobial agent sources, and could be used for various stored products, nosocomials, and crop-borne pathogens. Liu et al. [2] employed curcumin as antibacterial preservatives for edible coating using Pouteria campechiana pericarp waste as the substrate and carboxymethyl cellulose as the film-forming agent. The edible film obtained, thanks to the action of curcumin, showed excellent antibacterial properties, which can be exploited to preserve the quality of the food on which they are placed, increasing the storage time. Martínez-Abad et al. [84] applied a double-MAE process to lemon peels. In the first step, microwave-assisted hydrodistillation was performed to obtain lemon peel EOs, and then the biomass was treated with microwave-assisted extraction to recover lemon pigments. The obtained lemon essential oils showed a high antibacterial activity against *E. coli* and *S. aureus*, making them a valuable ingredient for functional food production.

Another important field of application for antibacterial molecules is given by their therapeutic use against microbial infections. El-Sawi et al. [67] found that extracts of potato peels showed a good cytotoxic activity against osteosarcoma cell line while all antibacterial showed moderate activities against the tested pathogens. This increased the likelihood for in vivo and subsequent clinical studies to assess the potential of natural products for the transformation of different therapeutic drugs against cancer as well as microbial infections in the near future. Lee et al. [10] used mandarin peels, considered an agri-food waste that could cause serious environmental pollution, to fabricate a functional bioelastomer with antioxidant and antibacterial activities. Bioactive compounds, extracted through MAE, were recovered from mandarin peels in liquid form and added to the bioelastomer during fabrication to maintain its mechanical strength. Bioelastomers obtained from extracts showed a higher mechanical strength than those obtained from biomass powder, and thanks to the use of the extracted additives, which show a high antioxidant and antibacterial activity, this allows their application in different industrial fields like pharmaceuticals and medical industries.

Finally, antimicrobial components of agri-food wastes are increasingly being used in cosmetics, due to their natural properties and sustainability. These compounds inhibit microbial growth, making them ideal for incorporation into skincare and hygiene products. They can improve the shelf life of cosmetics and provide additional benefits like anti-inflammatory and antioxidant effects, enhancing the overall quality and efficacy of formulations while reducing the reliance on synthetic chemicals. Kupnik et al. [30] identified and quantified different biologically active compounds from avocado seeds demonstrating their possible applications in cosmetics as well as biomedicine and pharmaceuticals. 

It is worthwhile to underline that antimicrobial molecules extracted from agri-food waste are very susceptible to degradation when exposed to light, humidity, or heat, causing the generation of free radicals that compromise their bioactivity [129]. For this reason, encapsulation has emerged as a viable solution to this problem allowing the controlled release of these compounds. Micro- and nanostructures have been explored as carriers for antimicrobial compounds with the aim of combating microbial attacks on crops (and on skin) and extending the shelf life of food products [130,131]. Many reviews in the literature report on the advantages of using this technique, the critical analysis of the different methodologies to carry out micro- and nanoencapsulation, and the principal types of encapsulation systems [130,132,133].

However, the application of nanoencapsulated antimicrobial molecules still meets some constraints that need to be developed further, such as application conditions, effectiveness studies, and industrial scale-up [130]. 

## 7. Bio-Economic Perspective

The bioeconomy focuses on harnessing renewable agro-waste biomass to sustainably generate food, energy, and industrial materials. Shifting from a fossil fuel-based economy to one centered on biological resources is expected to reduce the reliance on fossil fuels, promoting sustainability and environmental protection while simultaneously boosting the bioeconomy [134,135]. The transition from fossil to renewable resources will be possible only with an increased utilization of waste biomasses. In fact, the worldwide agri-food industry produces approximately 140 billion tons of waste biomass annually [136]. Agricultural wastes include by-products from fruit and vegetable processing, such as peels, seeds, and pulp as well as materials like crop residues, such as straw, husks, and stalks. Livestock farming generates waste through manure and animal by-products. In the food industry, waste comes from expired or damaged goods, kitchen scraps, and excess from food preparation. 

Nearly 40% of fruits and vegetables are thrown away post-harvest due to consumer preferences for visually flawless produce. Grocery stores favor flawless fruits and vegetables, as even slight imperfections can render a quality product unsellable. The industry that produces juice account for the production of a big amount of waste. As an example, in the orange juice industry, over 50% of the raw material turns into by-products, which are still rich in bioactive compounds and possess significant nutritional value. Also, according to the FAO’s 2023 report, Thailand exported nearly 339,917 tonnes of mangoes in 2022 [137]. During processing, fruit culls, peels, and seeds account for 35–60% of the total weight and are typically discarded as waste [138].

In this resource-constrained context, waste from grain processing also has an increasing appeal. In fact, the world was expected to use 2780 million tons of cereals in 2022/23, which is more than the total amount of cereals produced in 2022 [137,139]. In North America, Europe, and Asia, approximately 35% of total cereal production is wasted. Within industrialized Asia, cereal losses and waste account for up to 18% [140]. For these reasons, some efforts have been made towards creating functional materials, thus presenting a valuable opportunity in the context of a “zero waste” circular bioeconomy [12,141].

However, these wastes are transformed into compounds at very low levels. In most cases, the valorization of waste biomass is centered on developing bioprocesses to produce a single product. To ensure economic viability and sustainability, it is crucial to develop integrated processes that maximize resource use and to aim for a variety of end products that can meet diverse market needs [142]. Also, the creation of integrated biorefineries for producing biomaterials connect to the industries can create fresh market prospects for them.

Despite biomass waste potential for sustainable economic development and environmental benefits, there are some factors affecting its utilization as resource of renewable products. First, it is a variable material, making it difficult to standardize processes for its conversion into bioenergy or bioproducts. The efficient extraction of valuable compounds requires complex and advanced bioprocesses, which are still under development [143]. This requires significant initial investment and operational costs. Many processes are not yet economically competitive with fossil fuel-based alternatives, limiting widespread adoption.

Also, the agricultural waste is dispersed across large areas. This geographic spread creates logistical challenges for collection and transportation, adding to the overall costs and complexity of developing biomass valorization. Actually, the infrastructure needed to collect, transport, and process biomass waste on a large scale is lacking. Without the necessary logistics and processing facilities, it is challenging to build an efficient biomass valorization supply chain. Some regions in Canada, United States, India, Australia, and China have made significant strides in developing the infrastructure needed for large-scale biomass waste management, driven by a combination of policy support, technological innovation, and a focus on sustainability [144]. In Europe, only Germany, Sweden, and the Netherlands are known for innovative waste management practices [145]. For these reasons, further research is needed to develop cost-effective, scalable technologies for biomass valorization. Advancing extraction technologies to improve yield and efficiency is essential for unlocking the full potential of bioactive molecules. More consistent government policies and regulations around renewable energy and biomass valorization should be created. The market for biomass-based products and bioenergy is still emerging. Raising awareness and fostering public support are key factors to advancing this sector. At the time of this review, no commercial products with antimicrobial properties extracted from agri-food waste are available, nor are there any industrial-scale processes dedicated to the extraction of such bioactive compounds from agri-food waste. Furthermore, the number of patents in this area remains relatively limited when compared to the extensive volume of the scientific literature on the subject. Lazzi et al. [146] patented a specific extraction methodology for vegetable waste, which consists of the addition of a bacterium (Lactobacillus) with the aim of inducing a fermentation step before the extraction (Patent n. WO2020230044A1, 19 November 2020). At the end of fermentation, the fermented product can be directly subjected to extraction (in an aqueous and/or organic solvent) or be preserved by freezing. The extract comprises a mixture of compounds selected from polyphenols, organic acids (like acetic, citric and lactic acid), peptides, and amino acids and has demonstrated significant antimicrobial activity. Calderon Oliver et al. [147] patented a method for encapsulating compounds, with antimicrobial and antioxidant power extracted from avocado peel waste (Patent n. MX2017009114, 8 February 2019). These microparticles obtained from a colloidal aqueous solution by combining the extract with nisin, and are proposed for the preservation of meats. There are two other patents that are worthwhile to cite even if the antimicrobial activity of the compound is not clearly declared. The first one was deposited by Liu et al. [148] and reports an extraction method of an high-activity camellia nitidissima polyphenol substance, i.e., omega-hydroxpropioguaiacone, with high antioxidant and sterilizing effects (Patent n. CN118515542A, 20 August 2024). The second one is by Urrego Camelo et al. [149] and reports a method for producing encapsulated microparticles for the controlled release of a natural hydroalcoholic preservative obtained from walnut tree leaf and husk extracts (Patent n. WO2024227266, 26 April 2024).

## 8. Conclusions

Converting agri-food wastes into valuable products is essential, as improper disposal significantly affects the environment through high energy consumption, water use, and carbon emissions. This review has examined the most recent studies on extracting bioactive molecules with antimicrobial properties from both agri-food wastes and by-products and highlighted future opportunities to advance their production and processing in a circular economy perspective. An in-depth analysis of their extraction methods and antimicrobial activity against a broad range of bacteria has been reported and discussed. Additionally, potential mechanisms of action, which include the (i) disruption of bacterial cell membranes, (ii) inhibition of energy metabolism and bacterial DNA synthesis, and (iii) interference with quorum sensing and biofilm formation were reported.

These bioactive molecules offer diverse potential applications, from cosmetics to food packaging. In particular, they can be incorporated into food packaging materials to inhibit microbial growth, thereby enhancing food safety and shelf life of products. They can also be used as natural products for the transformation of different therapeutic drugs against cancer as well as microbial infections. Finally, the antimicrobial components of agri-food wastes are increasingly being used in cosmetics.

However, despite their promise, only a small fraction of waste is currently converted into valuable compounds. This limitation stems from high extraction costs, technical challenges, supply chain complexity, limited infrastructure, regulatory and policy obstacles, and public perception. Therefore, further research is essential to create cost-effective, scalable technologies for biomass valorization.
